# Trafficking of hormones and trophic factors to secretory and extracellular vesicles: a historical perspective and new hypothesis

**DOI:** 10.20517/evcna.2023.34

**Published:** 2023-11-09

**Authors:** Y. Peng Loh, Lan Xiao, Joshua J. Park

**Affiliations:** 1 Section on Cellular Neurobiology, Eunice Kennedy Shriver National Institute of Child Health and Human Development, National Institutes of Health, Bethesda, MD 20892, USA.; 2 Scientific Review Branch, National Institute on Aging, National Institutes of Health, Bethesda, MD 20892, USA.

**Keywords:** Hormone trafficking, trophic factor, neurons, endocrine cells, extracellular vesicles, sEV, exosomes

## Abstract

It is well known that peptide hormones and neurotrophic factors are intercellular messengers that are packaged into secretory vesicles in endocrine cells and neurons and released by exocytosis upon the stimulation of the cells in a calcium-dependent manner. These secreted molecules bind to membrane receptors, which then activate signal transduction pathways to mediate various endocrine/trophic functions. Recently, there is evidence that these molecules are also in extracellular vesicles, including small extracellular vesicles (sEVs), which appear to be taken up by recipient cells. This finding raised the hypothesis that they may have functions differentiated from their classical secretory hormone/neurotrophic factor actions. In this article, the historical perspective and updated mechanisms for the sorting and packaging of hormones and neurotrophic factors into secretory vesicles and their transport in these organelles for release at the plasma membrane are reviewed. In contrast, little is known about the packaging of hormones and neurotrophic factors into extracellular vesicles. One proposal is that these molecules could be sorted at the trans-Golgi network, which then buds to form Golgi-derived vesicles that can fuse to endosomes and subsequently form intraluminal vesicles. They are then taken up by multivesicular bodies to form extracellular vesicles, which are subsequently released. Other possible mechanisms for packaging RSP proteins into sEVs are discussed. We highlight some studies in the literature that suggest the dual vesicular pathways for the release of hormones and neurotrophic factors from the cell may have some physiological significance in intercellular communication.

## INTRODUCTION

Throughout evolution, from unicellular organisms to multicellular life forms, cells communicate via the secretion of signaling molecules packaged in vesicles which are organelles bounded by a lipid bilayer membrane^[1]^. The types of secreted vesicles and content vary with the type of cell and organism. Upon release, the contents of the vesicles perform various physiological functions^[2-6]^. Early studies towards understanding the secretory pathway in various cells were descriptive and morphological. In 1974, George Palade won the Nobel Prize for discovering, based on electron microscopic studies, that newly synthesized secretory proteins are transported vectorially from the endoplasmic reticulum to the Golgi apparatus and into secretory vesicles for release. Subsequently, the pioneering work of Randy Schekman exploiting yeast genetics transformed the secretion field into one that was molecular and mechanistic. Schekman, together with James Rothman and Thomas Sudhof received the Nobel Prize in Physiology or Medicine in 2013 for discovering the molecular mechanisms regulating intracellular vesicle trafficking along the secretory pathway (for review, see^[7]^). This chapter is for a Special Issue dedicated to honoring Randy Schekman. Although his discoveries stemmed from using yeast genetics, the proteins involved in membrane trafficking mechanisms are highly conserved in mammalian cells. Schekman’s findings revolutionized the secretion field from hormone secretion to cholesterol homeostasis and neurotransmitter release. Over the last decade, his work has inspired research in the field of membrane trafficking and vesicle biogenesis in the regulated and constitutive secretory pathways and, more recently, extracellular vesicle (EV) biogenesis, secretion and function in different cell types. This Special Issue will begin with our chapter, recapitulating some of the historical perspective and recent updates of the molecular mechanisms involved in the biogenesis, sorting, and packaging of regulated secretory pathway (RSP) proteins into classical secretory vesicles, and their intracellular trafficking and secretion in neuroendocrine cells. Each of the steps for the assembly of the RSP is highly orchestrated to ensure proper packaging of hormone and trophic factor precursors and proteolytic enzymes into secretory vesicles for processing to mature proteins/peptides necessary for intercellular communication and higher physiological function. Dysregulation of the RSP in (neuro)endocrine cells leads to various diseases. For example, improper sorting of proinsulin for processing to insulin results in type 2 diabetes^[8]^. Studies have implicated dysfunction of synaptic vesicle transport in the presynaptic terminals of dopaminergic neurons at the substantial nigra in Parkinson’s Disease^[9]^, and in Prader-Willi Syndrome, a decrease of secretory vesicle and neuropeptide production was found, leading to impaired hypothalamic neuroendocrine function^[10]^. Next, we highlight several sEV-related papers showing that some of these RSP proteins, including peptide hormones and neurotrophic factors, are also secreted as cargo in sEVs. We discuss the possible physiological significance of the dual release of these RSP proteins via classical secretory vesicles versus sEVs. Since sEVs are taken up by recipient cells, we hypothesize that these RSP proteins may have different biological functions intracellularly as opposed to their receptor-mediated signaling action extracellularly.

## SECRETORY PATHWAYS IN (NEURO) ENDOCRINE CELLS

**Figure 1.**
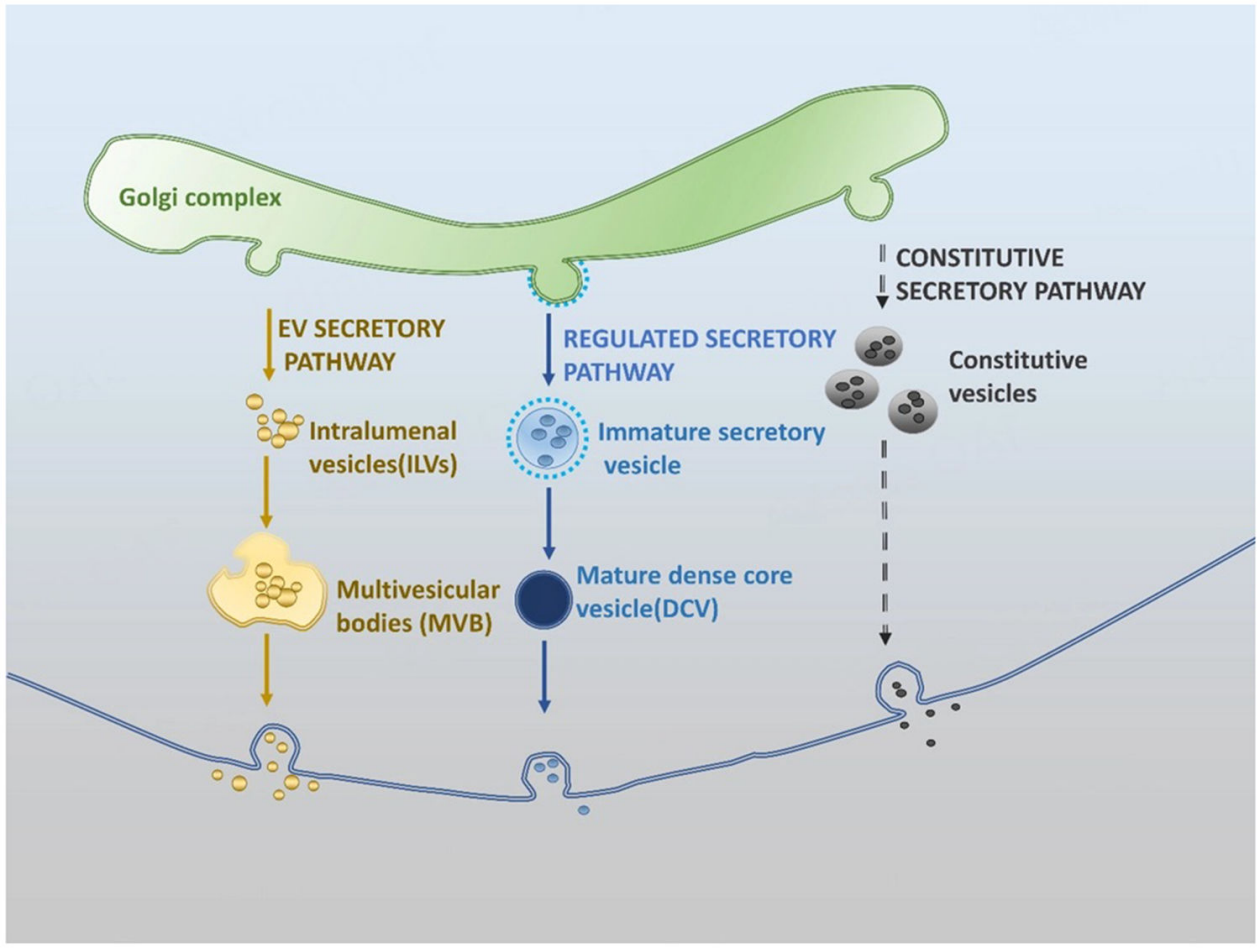
Secretory pathways in (neuro)endocrine cells. In endocrine cells and neurons, peptide hormones, neuropeptides, trophic factors, and granins are sorted at the TGN into immature vesicles that then mature to become DCV. Their content is released via the RSP upon stimulation. Other proteins in the Golgi complex are packaged into constitutive vesicles and secreted via the CSP, a default pathway. sEVs originate from ILVs formed through either endocytic pathway or possibly ER/Golgi secretory pathway. They are then taken up by MVBs. When the MVBs fuse with the plasma membrane, the sEVs are released. TGN: trans-Golgi network; DCV: dense core vesicles; RSP: regulated secretory pathway; CSP: constitutive secretory pathway; sEVs: small extracellular vesicles; ILVs: intraluminal vesicles; ER: endoplasmic reticulum; MVBs: multivesicular bodies.

In the classical RSP, endocrine and neuroendocrine cells package their hormones and trophic factors into dense core vesicles (DCVs) and release their content upon stimulation in a regulated manner, while other secretory proteins are released constitutively without stimulation [constitutive secretory pathway (CSP)]^[11]^ [Figure 1].

DCVs are 100-200 nm in size, whereas the constitutive vesicles are smaller (40-100 nm). The content of the DCVs is released by exocytosis to the extracellular space, and the secreted molecules exert their effects by binding to classical receptors or membrane protein-binding partners for receptor activation and downstream signaling. In endocrine cells, released hormones can be blood-borne and exert their effects by binding to receptors at some distance away from the release site. Conversely, in the nervous system, neurotrophic factors are released from RSP vesicles into the synaptic cleft, close to the proximity of the receptors on the post-synaptic terminals for their action. Recently, it has been found that neuroendocrine cells secrete sEVs. sEVs originate from intraluminal vesicles (ILVs) formed through either endocytic pathway or possibly endoplasmic reticulum (ER)/Golgi secretory pathway. They are then taken up by multivesicular bodies (MVBs), which fuse with the plasma membrane to release the sEVs [Figure 1]. sEVs, which are generally 30-100 nm in size, differ greatly in size from DCVs (100-200 nm).

## MECHANISMS OF PROTEIN SORTING AT THE TRANS-GOLGI NETWORK TO THE RSP

Regulated secretory proteins, which include peptide hormones and neurotrophins and their precursors, as well as granins, have a signal peptide at the N-terminus that directs them to the rough endoplasmic reticulum (RER) cisternae after synthesis. They are then transported to the Golgi complex, where they are sorted at the trans-Golgi network (TGN), prior to their entry to immature secretory vesicles. The hormones and neurotrophic precursors are proteolytically processed within the immature secretory vesicles [Figure 1]. These secretory vesicles then mature to become large (DCV, about 100-200 nm in diameter). The DCVs, which mainly include neurotrophins, neuropeptides or peptide hormones, are stored until stimulated to release. They release their contents slowly upon repeated stimulation or at low concentrations of calcium^[12,13]^, and are then replenished. Many reviews^[4,14-18]^ have been written about the mechanisms of sorting and packaging of peptide hormones and neurotrophins into the vesicles of the RSP. A brief summary of the RSP is presented in Figure 1. Here, we have highlighted examples of RSP protein sorting mechanisms. For the correct packaging of RSP proteins to (neuro)endocrine DCVs, there should be an elaborate process to sort RSP proteins to DCVs at the lumenal space of the TGN. The process is called Sorting-at-Entry and involves several mechanistic steps, including aggregation, the use of sorting scaffold/receptors, and structural motifs (e.g., disulfide bond)^[19,20]^.

**Figure 2.**
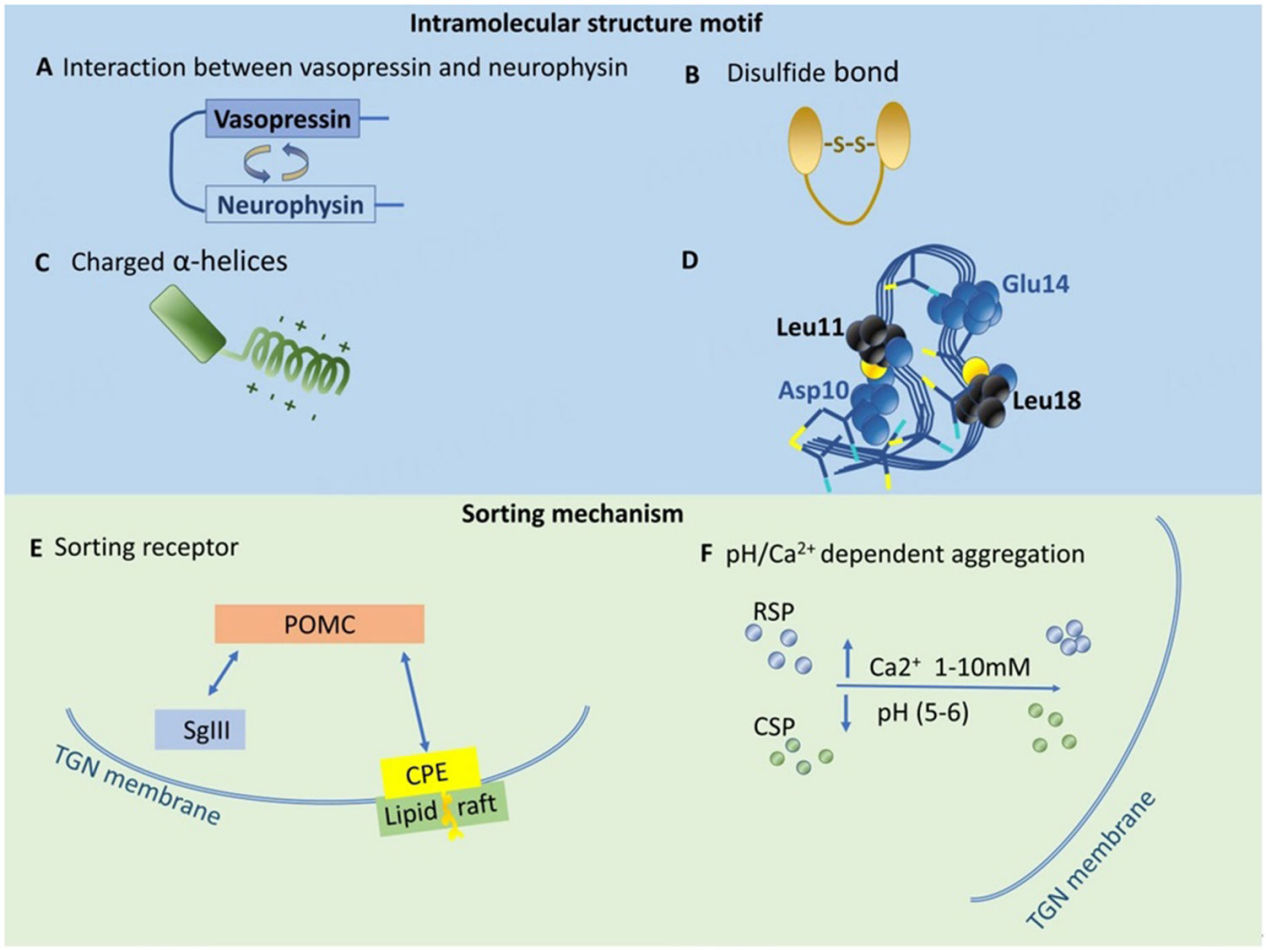
TGN lumenal sorting mechanisms. Motifs that are required for the sorting of RSP proteins to DCVs. These include (A) the interaction between vasopressin and neurophysin domains in their precursor form; (B) disulfide bond; (C) charged α-helices; (D) the sorting signal motif of POMC that is conformation-dependent and comprises of two acidic residues, Asp10 and Glu14, and the two hydrophobic residues, Leu11 and Leu18. (Figure reproduced from Cawley *et al.* with permission)^[27]^; (E) The sorting mechanism for POMC, pro-enkephalin, and BDNF use similar sorting motifs comprising of a pair of acidic amino acids binding to a pair of basic amino acids in a sorting receptor, membrane CPE which associates specifically with cholesterol-sphingolipid-rich lipid raft domains at the TGN membrane prior to budding off to form a DCV. (F) RSP proteins can also be sorted to the RSP by aggregation at pH 5-6 and 1-10 mM Ca^2+^ inside the TGN lumen. TGN: trans-Golgi network; POMC: pro-opiomelanocortin; BDNF: brain-derived neurotrophic factor; CPE: carboxypeptidase E; DCV: dense core vesicles.

Several types of structural motifs have been identified to be required for the sorting of various RSP proteins to DCVs [Figure 2]. For the sorting of vasopressin to the RSP, the interaction between vasopressin and neurophysin domains in their precursor protein is required for the sorting of vasopressin into DCVs [Figure 2A]. The inhibition of the interaction by either deletion or mutation of the neurophysin-interacting domain of vasopressin blocks the sorting of vasopressin to DCVs in Neuro2A cells and accumulates their precursor proteins in the ER^[21]^. For Chromogranin B (CgB), its N-terminal disulfide bond is needed for sorting to the RSP^[22,23]^ [Figure 2B]. Some prohormones including proglucagon [Figure 2C]^[24]^, the precursors of cocaine and amphetamine-regulated transcript (CART) peptide^[25]^, and dopamine neurotrophic factor^[26]^ require a charged α-helix domain for their sorting to the RSP.

Another type of sorting motif has been identified for pro-opiomelanocortin (POMC), pro-enkephalin, and brain-derived neurotrophic factor (BDNF). The sorting motif consists of a pair of acidic amino acids that bind to a pair of basic amino acids of the sorting receptor, membrane carboxypeptidase E (CPE), which is specifically associated with cholesterol-sphingolipid-rich lipid raft domains at the TGN membrane [Figure 2D]. The complex of either prohormone (POMC)-CPE or proBDNF-CPE then buds from the TGN to form DCV^[17,28-34]^ [Figure 2E]. In addition, the cytoplasmic tails of transmembrane proteins in DCVs also play a role in the sorting of RSP proteins to DCVs. For example, the cytoplasmic tails of vesicular monoamine transporters (VMATs) contain a sorting signal to RSP^[35]^. Two glutamate residues upstream of dileucine-like motif in the cytoplasmic tail of VMATs are required for their sorting to RSP. If the glutamate residues are mutated to alanine residues, the sorting of VMATs to DCVs is reduced.

Although there is evidence for RSP sorting motifs on some studied prohormones, it has been proposed that RSP proteins may also be sorted by aggregation with granins. Many RSP proteins aggregate at pH 5-6 and 1-10 mM Ca^2+^ in the TGN lumen^[36]^. Secretogranin II (SgII) is a granin that aggregates in a pH-dependent manner, thus driving DCV formation at the TGN^[37]^ [Figure 2F]. The deletion of SgII causes not only the reduction of the number and size of DCVs but also the trafficking of other hormones to DCVs in PC12 cells. In secretion-deficient PC12 cells, the expression of SgII restores the regulated secretion of hormones. Exocrine pancreatic RSP proteins are also aggregated in the condition mimicking the TGN lumen that facilitates sorting to secretory vesicles^[38]^. Secretogranin III (SgIII), another granin, was shown to bind POMC to facilitate the sorting of POMC to the RSP. It was proposed that the SgIII-POMC complex is then transferred to Chromogranin A (CgA) to facilitate their sorting to the DCVs [Figure 2E]^[39]^.

## PROTEIN PACKAGING AND DCV FORMATION AT THE TGN IN THE RSP

### Proteins that mediate DCV formation at the interface between the lumen and the cytoplasm

Secretory vesicles are formed at the cholesterol-sphingolipid-rich membrane domains of the TGN by reverse pinocytosis. CgA is an important molecule in driving DCV formation (for review, see^[15]^). When CgA was deleted in PC12 cells^[40]^ and mice^[41]^, the number of DCVs was significantly decreased due to the degradation of granular proteins. On the other hand, the expression of CgA in non-secretory cells induced the formation of DCV-like vesicles^[40]^. However, the DCV-forming function of CgA appears to be cell type-specific since the formation of insulin-containing DCVs formation is entirely independent of CgA^[42,43]^. It appears that other granins compensate for the loss of CgA in DCV formation^[44,45]^.

**Figure 3.**
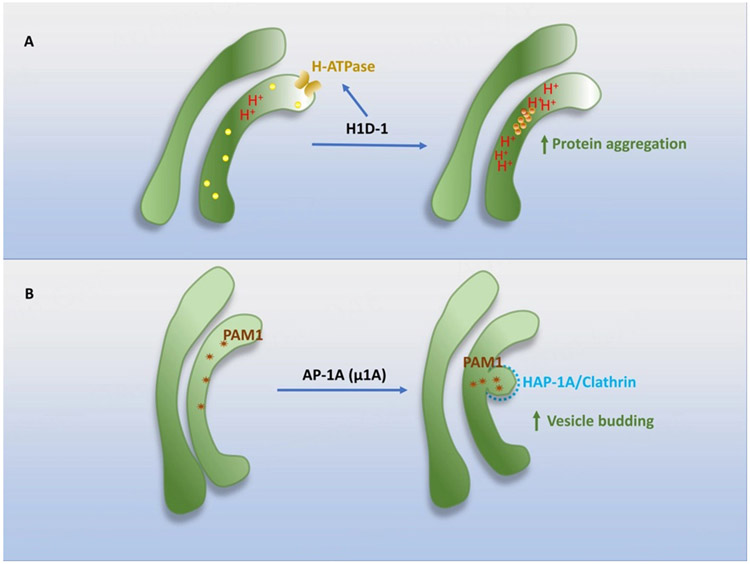
Cytoplasmic contribution to DCV formation (I). (A) HID-1 promotes the acidification of TGN lumen by increasing H-ATPase activity, subsequently decreasing pH value and facilitating protein aggregation; (B) The subunit of AP-1A complex is critical for the sorting of PAM-1 to immature DCVs from TGN. Proteins (yellow), Protein Aggregates (orange), PAM1 (red). HID-1: high-temperature-induced dauer formation protein 1; AP-1A: adaptor protein 1A; PAM-1: peptidylglycine α-amidating monooxygenase-1; TGN: trans-Golgi network; DCV: dense core vesicles.

Some TGN lumenal membrane proteins seem to facilitate the formation of DCVs [Figure 3]. One of them is the peripheral TGN membrane protein, High-temperature-induced dauer formation protein 1 (HID-1). The knockout of HID-1 was reported to inhibit the regulated secretion of insulin in pancreatic β cells upon its knockout in mice^[46]^ and reduce the levels of DCV cargo proteins in C. elegans^[47,48]^. One recent study showed that HID-1 knockout in PC12 cells blocked the acidification of TGN lumen by the mis-localization of the Golgi-targeted H-ATPase subunit 2^[49]^. Thus, it appears that HID-1 drives DCV formation by facilitating the acidification of TGN lumen for the pH-dependent aggregation of RSP-targeted hormones [Figure 3A].

One player involved in DCV formation at the interface is Adaptor protein 1A (AP-1A) complex. The μ1A subunit of the complex is crucial for the sorting of two prohormone processing enzymes, carboxypeptidase D (CPD) and peptidylglycine α-amidating monooxygenase-1 (PAM-1), to immature DCVs from the TGN^[50]^. The reduction of the μ1A subunit decreased the number of immature DCVs and caused the appearance of RSP proteins in non-DCV vesicles. The AP-1A-mediated formation of DCVs appears to be mediated by the interaction of the cytoplasmic tail of PAM-1 with AP-1A [Figure 3B].

### Mechanisms for DCV formation at the cytoplasmic side of the TGN

**Figure 4.**
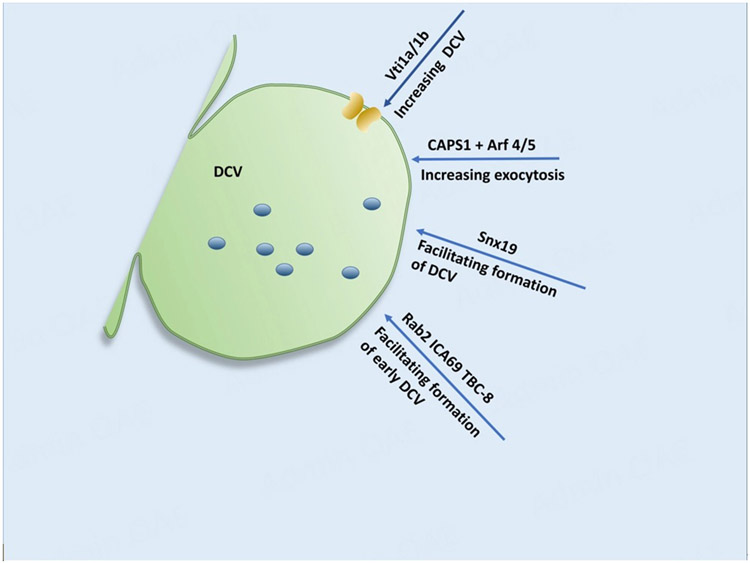
Cytoplasmic contribution to DCV formation (II). SNARE and t-SNAREs 1A/1B (Vti1a/1b) are involved in the formation of DCV. Vti1a also plays a role in DCV generation and Ca^2+^ channel trafficking. CAPS1 regulates the exocytosis of DCVs. Arf4/Arf5 is involved in CAPS1-mediated DCV formation. Snx19 is required for the formation of DCVs. Rab2, along with ICA69 and TBC-8, are involved in the early DCV formation. PICK1 is involved in the ICA69-mediated DCV formation from endosomal origin. Proteins (blue); SNARE: soluble N-ethylmaleimide-sensitive factor attachment protein receptor; CAPS1: Ca^2+^-dependent activator protein for secretion 1; Arf4: ADP-ribosylation factor 4; Snx19: sorting nexin 19; ICA69: islet cell autoantigen of 69 kD; TBC-8: Tre-2/Bub2/Cdc16 protein 8; PICK1: protein interacting with C-kinase 1.

A group of cytoplasmic proteins play a role in mediating the formation of DCVs at the cytoplasmic side of the TGN [Figure 4]. In neurons, Vesicle transport through interaction with t-SNARE 1A/1B (Vti1a/1b) mediates the formation of DCV^[51]^. Upon the loss of Vti1a/1b, the transport of DCV cargo into the axon was decreased, empty vesicles were accumulated, and the Golgi cisternae were distended. This suggests that Vti1a/1b is required for the formation of DCVs in neurons. Vti1a also appears to play a role in Ca^2+^ channel trafficking to the plasma membrane in chromaffin cells for DCV formation^[52]^. In the chromaffin cells, Vti1a was found at the TGN but not in mature DCVs. Upon the knockout of Vti1a, there were fewer secretory vesicles of reduced size and fewer Ca^2+^ channels at the plasma membrane without any effect on the release kinetics of Ca^2+^ channels.

Ca^2+^-dependent activator protein for secretion 1 (CAPS1) that regulates the exocytosis of DCVs at the secretion sites of the nerve terminals^[53,54]^ was found to be involved in DCV formation at the TGN via the interaction of its pleckstrin homology (PH) domain with ADP-ribosylation factor 4 (Arf4)/Arf5 in a GDP-dependent manner in neuroendocrine cells^[55]^. The knockdown of CAPS1 caused the accumulation of chromogranin in the Golgi complex and reduced the secretion of DCVs. Similarly, the overexpression of Arf5 mutants deficient in CAPS1-binding did the same, indicating that Arf5 is involved in CAPS1-mediated DCV formation. In addition, CAPS1 appears to play a role in DCV formation in SgII- and BDNF-containing DCVs in the brain^[56]^. In forebrain-specific CAPS1 knockout mice, the protein levels of SgII and syntaxin 6 (Stx6) were decreased, resulting in reduced presynaptic DCVs and dilated TGN cisternae. In cerebellum-specific CAPS1 knockout, the protein levels of SgII and BDNF were reduced and the number of DCVs at the fiber-Purkinje synapses was decreased significantly. These findings point to the significant role of CAPS1 in DCV formation in the neurons.

Sorting nexin 19 (Snx19) appears to regulate insulin secretion and DCV formation via its interaction with insulinoma-associated protein 2 (IA-2) in mouse pancreatic β-cells^[57]^. In pancreatic MIN6 cells, the knockdown of Snx19 not only reduced insulin content and secretion but also significantly decreased the number and size of DCVs. The reintroduction of Snx19 into Snx19 knockdown cells reversed the abnormal insulin secretion and DCV formation. This suggests that Snx19 is required for the formation of insulin-containing DCVs in pancreatic β-cells. Rab2 also plays a major role in DCV formation at the cytoplasmic side of the TGN. The collaboration of Rab2 with islet cell autoantigen of 69 kD (ICA69) and its GTPase activating protein, Tre-2/Bub2/Cdc16 protein 8 (TBC-8), was found to be required for early DCV formation at TGN [Figure 4]^[58-62]^.

Interestingly, microtubules also appear to be involved in DCV formation at the TGN, namely Golgi-derived microtubules (GDMTs). In pancreatic β cells, the prevention of microtubule nucleation around the Golgi complex drastically inhibited the exit of proinsulin from the TGN, resulting in the accumulation of proinsulin in the Golgi cisternae^[63]^. This report proposes that the biogenesis of DCVs at the TGN requires microtubule nucleation for the generation of a GDMT network around DCV-forming sites.

## MECHANISM FOR SORTING OF RSP PROTEINS INTO DCV BY POST-GOLGI RETENTION

**Figure 5.**
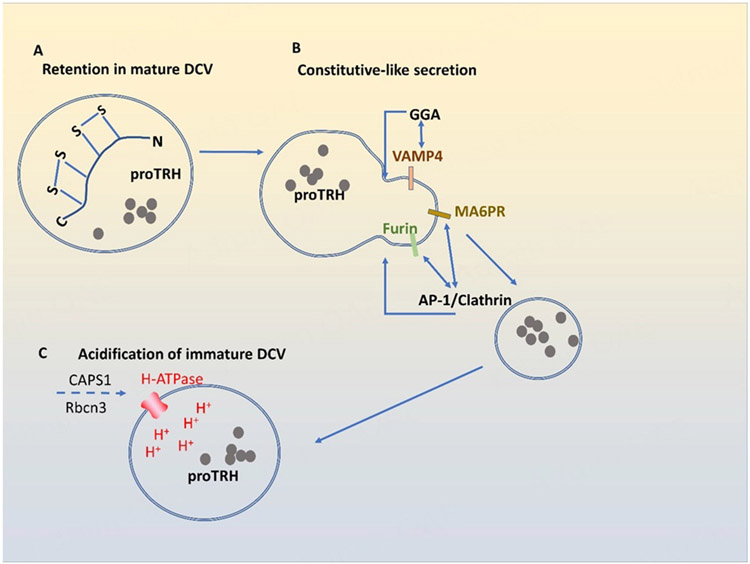
Sorting of RSP proteins into DCV by retention and constitutive-like secretion. RSP proteins can be packaged into DCVs by a “sorting-by-retention” mechanism: C-terminal disulfide bond is necessary for proTRH to remain in RSP vesicles (A); Non-RSP proteins in immature DCVs are removed by constitutive-like secretory pathways. AP-1 binds to the cytoplasmic tails of furin and M6PR and removes furin and M6PR from immature DCV via clathrin-mediated constitutive-like secretion (B). Golgi-localized, γ-ear containing ADP-ribosylation factor binding (GGA) mediates the removal of VAMP4 from immature DCVs (B); APS1 increases the activity of H-ATPase on DCVs to facilitate vesicle acidification. Rbcn3 promotes the translocation of CAPS1 to DCVs from the cytoplasm (C). proTRH: prothyrotropin-releasing hormone; M6PR: mannose-6-phosphate receptor; Rbcn3: rabconnectin 3; DCV: dense core vesicles; RSP: regulated secretory pathway.

Sorting-by-Retention is proposed as a mechanism for retaining RSP proteins in DCVs during DCV maturation in post-Golgi trafficking^[19,20,64]^. Such a mechanism was shown to retain pro-thyrotropin-releasing hormone (proTRH) in DCVs [Figure 5]. One intramolecular disulfide bond in the carboxy-terminal domain of proTRH is required for the retention of this prohormone in DCVs^[65]^. The mutation of two cysteine residues involved in the disulfide bond to glycines increased the constitutive secretion of proTRH and caused the defective processing of pro-TRH in endocrine cells such as AtT20 cells. Given that both mutant and wild-type proTRHs colocalize in RSP vesicles, the disulfide bond of proTRH appears to function as a motif to retain proTRH inside maturing DCVs [Figure 5A].

Non-RSP proteins in immature DCVs are removed by constitutive-like secretory pathway [Figure 5B]^[66]^. Clathrin, AP-1, and Golgi-localized, γ-ear containing, ADP-ribosylation factor binding (GGA) play a major role in the constitutive-like secretion. AP-1 binds to the cytoplasmic tails of furin and mannose-6-phosphate receptor (M6PR) on immature DCVs and removes furin and M6PR from immature DCVs via clathrin-mediated budding-off^[64,67-70]^. Conversely, GGA mediates the removal of vesicle-associated membrane protein 4 (VAMP4) from immature DCVs in PC12 cells^[71]^. After constitutive-like secretion, immature DCVs become more mature with a denser core.

## ACIDIFICATION AND OTHER STEPS INVOLVED IN DCV MATURATION

Immature DCVs undergo acidification during maturation. CAPS1 involved in DCV formation at the TGN also plays a role in DCV maturation by increasing the activity of vacuolar H-ATPase on DCVs for vesicle acidification [Figure 5C]. In human neuroendocrine BON cells, the rabconnectin 3 (Rbcn3) complex consisting of Dmx-like2 (DMXL2) and WD repeat domain 7 (WDR7) proteins recruits CAPS1 to DCVs from the cytoplasm^[72]^. The knockdown of either Rbcn3 or WDR7 caused the dissociation of CAPS1 from DCVs, while the knockdown of CAPS1, Rbcn3, or WDR7 impaired the acidification of DCVs. Thus, it appears that CAPS1 recruited by Rbcn3 complex onto DCVs facilitates the H-ATPase-mediated acidification of DCVs.

Besides acidification, CD63, a lysosome-related organelle (LRO)-associated protein, was found to be involved in DCV maturation^[73]^. The CD-63-mediated DCV maturation depends on the type II phosphatidylinositol 4 kinase (PI4KII)-dependent accumulation of phosphatidylinositol 4 phosphate (PI4P) on DCVs^[73]^.

## MICROTUBULE-BASED TRANSPORT OF DCVS TOWARDS THE PLASMA MEMBRANE REGION

**Figure 6.**
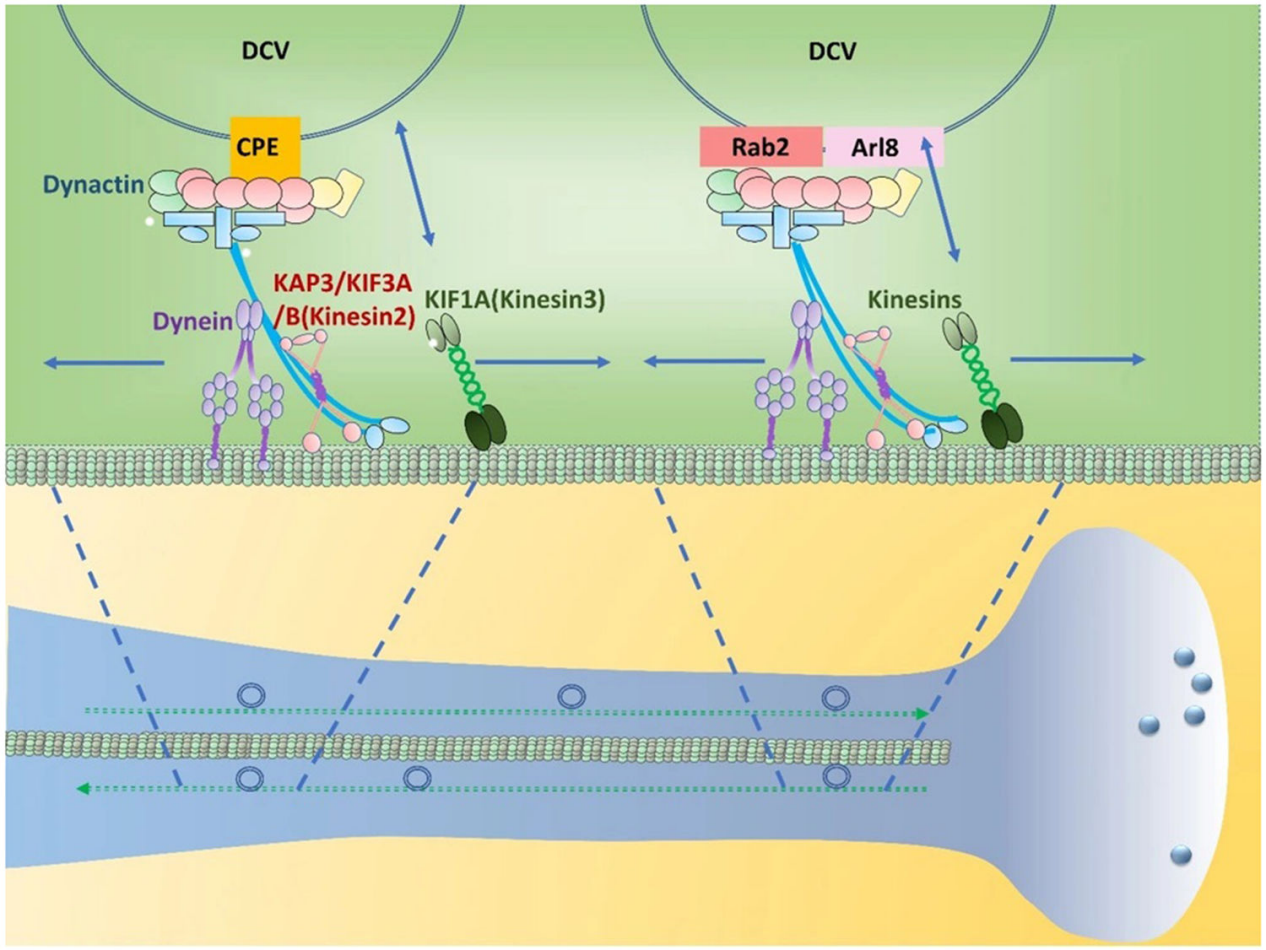
Microtubule-based bidirectional DCV transport. The anterograde axonal transport of DCVs on microtubules is mediated mainly by kinesin-3 and some by other kinesins. Rab2 is required for bidirectional DCV transport in the axon. The Arf-like GTPase, Arl8, is an adaptor for kinesin-3-mediated DCV movement. CPE cytoplasmic tail on DCVs recruits dynactin and KIF1A (kinesin-3) and KIF3A (kinesin-2). KIF1A and KIF3A mediate the anterograde transport of these vesicles on microtubules. Cytoplasmic dynein, a minus-end directed motor, binds dynactin and mediates the return of DCVs from the neurite terminus back to the cell body under non-stimulated conditions for vesicle homeostasis. Dynactin, a microtubule anchor protein complex for cytoplasmic dynein and kinesins, mediates the bidirectional movement of DCVs. CPE: carboxypeptidase E; DCV: dense core vesicles.

DCVs are transported from the TGN to the plasma membrane via microtubule-based anterograde transport. The anterograde transport of DCVs on microtubules along neuronal axons appears to be mediated mainly by kinesin-3 and some by other kinesins [Figure 6]^[74-77]^. In hippocampal neurons, kinesin-3 is the primary anterograde transporter of DCVs^[77]^. The involvement of kinesin-3 in DCV transport is also documented in the anterograde transport of POMC in the anterior pituitary cells and BDNF in mouse hippocampal neurons^[78,79]^. The interaction between the cytoplasmic tail of CPE and the microtubule motor complex containing kinesin-3 appears to be involved in the anterograde DCV transport in endocrine cells and neurons [Figure 6].

Rab2 involved in DCV formation at the TGN also plays a role in axonal DCV transport in Drosophila neurons^[80]^. In the neurons, Rab2 is required for bidirectional DCV transport in the axon, but not in the cell body, and found at the nanometer-range proximity to kinesin-3 [UNC-104: kinesin family protein 1A (KIF1A)]. The Arf-like GTPase, Arl8, that showed a similar inhibitory effect to Rab2 knockout on the bidirectional axonal DCV transport^[80]^ appears to be an adaptor for kinesin-3-mediated DCV movement^[81]^ and mediate the exit of DCVs from the cell body to the axon^[80]^ [Figure 6]. End Binding protein 1 (EBP-1) is also involved in the interaction between UNC-104 (KIF1A) and DCVs. EBP-1 was shown to promote the delivery of DCVs to the axons of C. elegans neurons^[82]^. EBP-1 mutations in C. elegans neurons significantly inhibited not only the exit of DCVs from the cell body to the axon but also the axonal secretion of DCVs. EBP-1 appears to start working at the TGN to enrich UNC-104/KIF1A near DCV sorting sites via its interaction with both kinesin-3 and microtubules for axonal DCV delivery.

## MECHANISMS REGULATING PROCESSIVITY AND POLARITY OF MICROTUBULE-BASED DCV TRANSPORT

Thus far, several potential mechanisms have been proposed to control the microtubule-based transport of DCVs. One mechanism appears to be phosphorylation-dependent regulation. For example, the c-jun N-terminal kinase (JNK) was found to mediate the serine phosphorylation of syntaxin 4 (Stx4), thus disconnecting kinesin-3 (KIF1A) from Stx4 on DCVs and releasing DCVs to the F-actin meshwork at presynaptic boutons^[83]^. The JNK-mediated Stx4 phosphorylation appears to be synaptic activity-dependent. On the other hand, the polarity of the bidirectional microtubule-based movements of DCVs is also affected by phosphorylation. In mouse hypothalamic oxytocin neurons, protein kinase A (PKA) and protein kinase C (PKC) seem to control the polarity of the microtubule-based transport of oxytocin vesicles^[84]^. Upon PKA activation by forskolin, the anterograde transport of oxytocin DCVs was greatly enhanced while it was blocked by PKC activation. In line with this observation, PKA activation increased the binding of kinesin-3 and kinesin-2 to Annexin 1A on DCVs, which should increase the anterograde transport. On the other hand, PKC activation reduced the binding of kinesin-3 to Annexin 1A on DCVs, which should decrease the anterograde transport.

In C. elegans, the axonal anterograde transport of DCVs in cholinergic motor neurons depends on cyclin-dependent kinase 5 (CDK5) and its activator, CDKA-1/p35^[85]^. In cdk5 or cdka-1/p35 mutants, DCVs were never transported into the axons but accumulated in the dendrites. Given that the axonal microtubules have a clear polarity with their plus ends towards the axonal terminal and minus ends towards the cell body, the absence of CDK5 activity appears to either block the anterograde transport of DCVs towards the plus ends of microtubules or enhance the retrograde transport of DCVs. To differentiate the two different possibilities, the loss-of-function mutation of cytoplasmic dynein was introduced into the cdk5 mutant. The double mutations blocked only the accumulation of DCVs in the dendrites where microtubule minus ends are mixed with their plus ends regarding orientation. Based on the results, this study speculates that CDK5 enhances cytoplasmic dynein-mediated retrograde DCV transport in both axon and dendrite [Figure 6].

Dynactin, a microtubule anchor protein complex for cytoplasmic dynein and some kinesins, was reported to mediate the bidirectional movement of DCVs in anterior pituitary cells and hippocampal neurons^[78,79]^ [Figure 6]. In a recent study, DCVs containing BDNF, neuropeptide Y (NPY), and tissue plasminogen activator (tPA) showed different polarities during microtubule-based transport in the axons and dendrites in hippocampal neurons^[86]^. When the dynactin complex was disrupted by the overexpression of p50 (dynamitin) that links the p150 side arm of dynactin to its base Actin-related protein 1 (Arp1) filament, the bidirectional movements of DCVs along the axon and dendrites were reduced. Conversely, the overexpression of p150 coiled-coil domain 1 (CC1), where cytoplasmic dynein binds, inhibited only motor movement processivity. Thus, it appears that the interaction of cytoplasmic dynein with dynactin may enhance the processivity of retrograde transport of the DCVs. This result confirms that dynactin is involved in the bidirectional movement of DCVs along microtubules in the axons and dendrites of hippocampal neurons [Figure 6].

Myosin Va, a F-actin-based motor protein, was also implicated in the regulation of the polarity of the bidirectional microtubule-based movement of DCVs. In cultured hippocampal neurons, the expression of the dominant negative tail construct of myosin Va reduced the velocity of the retrograde transport of large DCVs in the axon, while the axonal anterograde DCV transport was not affected^[87]^. This suggests that myosin Va facilitates the retrograde transport of DCVs along the microtubules.

## ACTIN-MEDIATED TETHERING REGULATES TRANSPORT AND SECRETION OF DCVS AT THE PERI-PLASMA MEMBRANE REGION

**Figure 7.**
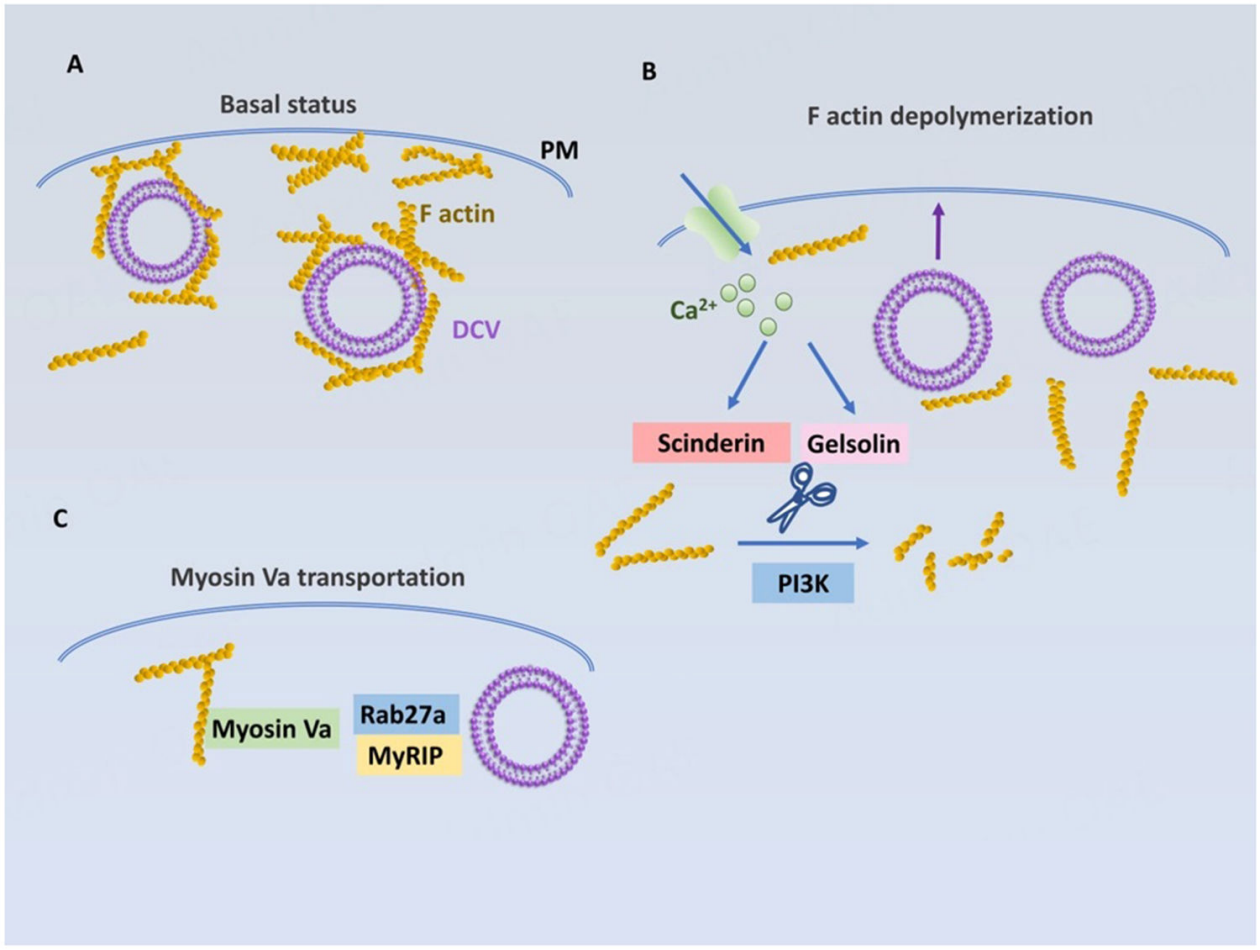
F-actin network controls DCV tethering, transport and secretion at the plasma membrane. (A) F-actins block the access of DCVs to the PM; (B) Actin-severing proteins such as scinderin and gelsolin cut F actins into small filaments, thus facilitating the release of DCV. PI3K causes the depolymerization of F-actins to facilitate the docking of DCVs to the PM; (C) Myosin Va is activated by increased Ca^2+^ levels during stimulated secretion and interacts with Rab27a and MyRIP on DCVs to facilitate the mobilization of DCVs to the PM. PM: plasma membrane; PI3K: phosphatidylinositol 3 kinase; DCV: dense core vesicles.

F-actin meshwork at the peri-plasma membrane region plays an important role in storing DCVs for regulated secretion [Figure 7A]. F-actins are thought to block the uncontrolled access of DCVs to the plasma membrane. The depolymerization of F-actins increases the secretion of RSP hormones^[88,89]^ while their stabilization suppresses Ca^2+^-stimulated hormone secretion^[88,90]^. Nonetheless, for stimulated exocytosis, DCVs should be released from the F-actin meshwork. Some actin-severing proteins are proposed to mediate the release. Scinderin is one of the actin-severing proteins that release mucin vesicles in airway cells^[91]^ and insulin vesicles in pancreatic β cells^[92]^. The other F-actin-severing protein, gelsolin, also affects amylase release in pancreatic acinar cells^[93]^ and insulin secretion in pancreatic β cells^[94]^. In addition, phosphatidylinositol 3 kinase (PI3K) appears to partake in the depolymerization of F-actin meshwork to facilitate the docking of DCVs to the plasma membrane^[95]^. Upon the knockdown or inhibition of p110γ, a subunit of PI3K, the cortical F-actin meshwork was increased while the docking of DCVs to the plasma membrane was decreased. This resulted in a reduction in Ca^2+^-stimulated insulin secretion [Figure 7B].

Of note, although the major function of F-actins is to tether DCVs at the peri-plasma membrane region, the extensive depolymerization of F-actins actually blocks the exocytosis of DCVs^[96,97]^. Hence, some F-actin filaments should remain to provide DCV transport to the plasma membrane. The F-actin-based DCV transport is mediated by the F-actin-based motor, Myosin Va. Myosin Va was found to be associated with DCVs^[98]^, melanosomes^[99]^, chromaffin granules^[100]^, and insulin vesicles^[101]^. The interaction of Myosin Va with DCVs appears to be mediated by Rab27a and MyRIP [Figure 7C]^[102-104]^. It was also found that Myosin Va is activated by increased Ca^2+^ levels, apparently contributing to stimulated DCV release^[105]^. Thus, the F-Actin network plays a critical role in the storage and mobilization of DCVs at the plasma membrane region, which ultimately regulates the final step of secretion of these vesicles in a calcium-dependent manner.

## HORMONE AND NEUROTROPHIC FACTORS ARE TRAFFICKED TO EXTRACELLULAR VESICLES

Besides classical RSP and constitutive secretory vesicles, another class of secretory vesicles known as EVs have been described in 1987^[106]^. These EVs are of different sizes, and ones of 30-150 nm in diameter have been referred to as sEVs or exosomes. Initially, sEVs were thought to be a means of removing waste from cells. sEVs have been found to be released from all tissues in animals, yeast, and bacteria, where these vesicles are known as outer membrane vesicles. These sEVs contain protein, RNA, DNA, and enzymes, and their contents reflect the physiology and pathophysiology of the original parental cell. Their membranes are enriched in tetraspanins^[107]^ and are therefore quite different from DCVs. Recently, sEVs have garnished much attention as novel intercellular messengers^[108]^. The biogenesis and secretion of sEVs differ from DCVs. The biogenesis of sEVs occurs when intraluminal vesicles formed from either endocytic pathway or endoplasmic reticulum (ER)/Golgi secretory pathway are taken up by MVBs. Then, the MVBs fuse with the plasma membrane to release the sEVs. The released sEVs are taken up by recipient cells, and the cargos in the sEVs are released into the cytoplasm to exert their physiological effects^[109]^. Recently, hormones, growth factors, and neurotrophins, normally present in classical RSP secretory vesicles, have been found in sEVs as well. RSP proteins such as parathyroid hormone-related protein (PTHrP)^[110]^, pro-BDNF and mature BDNF^[111]^, CPE/neurotrophic factor-α1 (CPE/NF-α1)^[112]^, several granins (e.g., CGA^[113]^, Secretogranin III^[114]^, and Neurogranin^[115]^), and amyloid precursor protein (APP)-derived Aβ peptides^[109,116]^ were reported to be present in sEVs. Such proteins are normally trafficked from the RER to the TGN, packaged into RSP DCVs, and released by exocytosis as described above [Figure 1]. However, the mechanism of routing such RSP proteins into sEVs is unclear. Moreover, the functional significance of hormones and neurotrophic factors in sEVs remains to be explored. Some studies on the RSP protein, CPE/NF-α1, suggest that release via the two mechanisms, from classical secretory vesicles (DCVs) as soluble molecules, or within sEVs which are taken up by recipient cells, may have different functions^[112,117]^.

### CPE/NF-α1

CPE is a prohormone processing enzyme expressed in endocrine cells and neurons, as well as in many cancer cells^[118-120]^. It also has neurotrophic activity and was given an alternative name, NF-α1^[121]^. CPE/NF-α1 is a RSP protein that has many non-enzymatic roles. Its transmembrane form acts as a sorting receptor at the TGN to target prohormones to the RSP vesicles^[34]^. Within the secretory vesicle, soluble CPE acts as a prohormone processing enzyme. The transmembrane form of CPE has a cytoplasmic tail that interacts with microtubule motors to transport the vesicles to the release site^[122]^. CPE mediates intercellular communication upon secretion from RSP vesicles by binding to the human membrane serotonin receptor, HTR1E, to activate the extracellular signal-regulated kinase (ERK)-B-cell lymphoma 2 (BCL2) signaling pathway to promote cell survival in neurons and cancer cells^[117,123,124]^. Interestingly, CPE and *Cpe* mRNA have been found in sEVs in many types of cancer cells, including human hepatocellular carcinoma (HCC) cells^[112]^. *Cpe* mRNA level in serum-derived, immunoaffinity purified sEVs from cancer patients with various types of cancer has been shown to be correlated with the metastatic state of the disease. Hence, sEV CPE is a potential prognostic cancer biomarker^[112]^.

**Figure 8.**
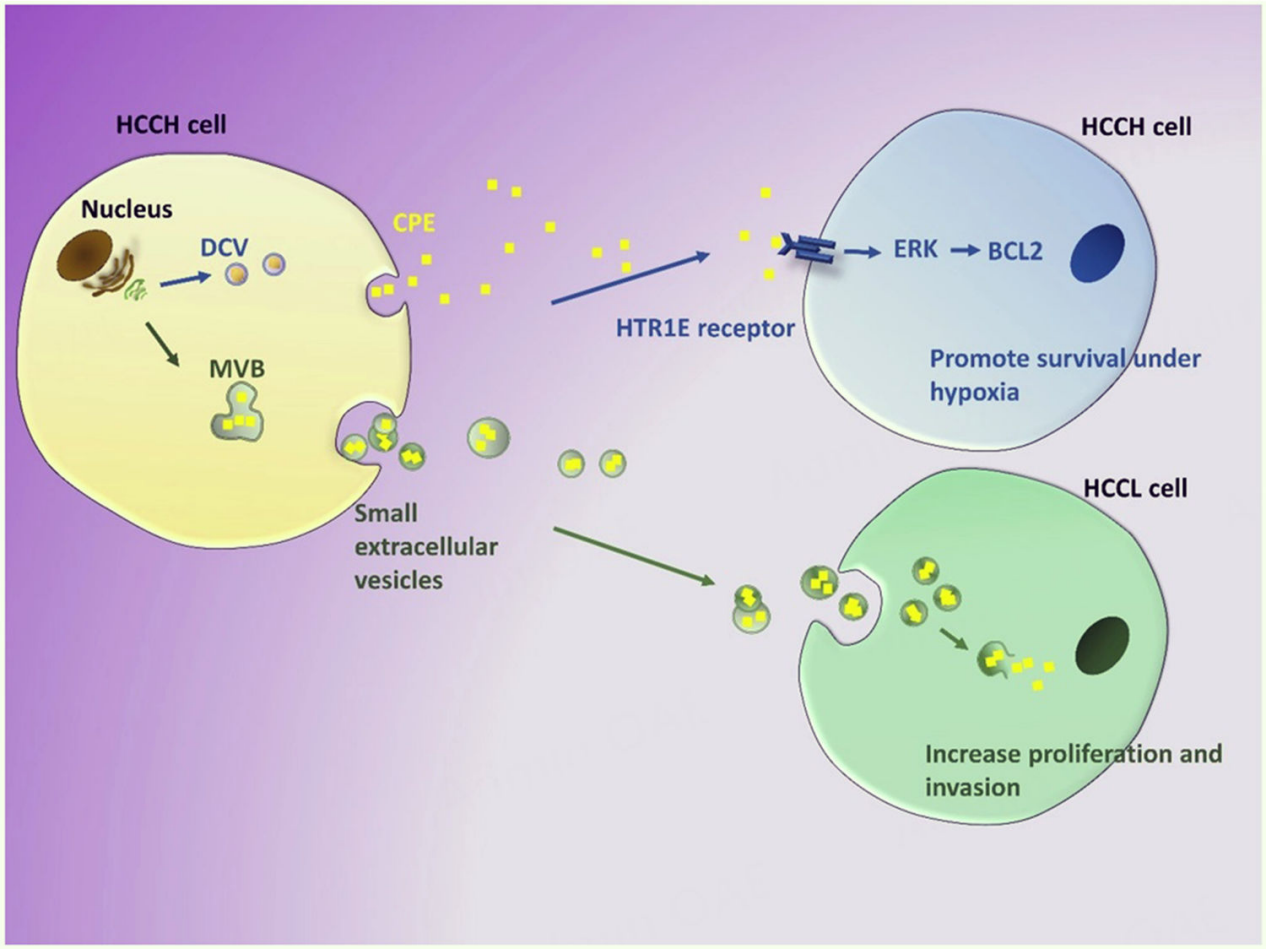
Different biological activities of DCV-derived soluble CPE and sEV-CPE. Soluble CPE secreted from high metastatic HCCH cells inds HTR1E receptors on recipient HCCH cells and activates ERK-BCL2 signaling pathway to promote survival under hypoxic stress. HCCH-derived sEVs that contain CPE are taken up by recipient low metastatic HCCL cells to enhance proliferation and invasion. HCCH: hepatocellular carcinoma; DCV: dense core vesicles; sEVs: small extracellular vesicles; CPE: carboxypeptidase E.

Studies have been carried out on PC12 (a pheochromocytoma neuroendocrine cell line) and HCC cells to examine the effect of soluble CPE, secreted from secretory vesicles into the cell culture medium, or exogenously applied, on the proliferation and survival of these cells. Secreted CPE and exogenously applied CPE promoted survival of pheochromocytoma (PC12) cells under nutrient starvation, and HCC cells under hypoxic conditions, respectively. HCC cells treated with CPE under hypoxic conditions activated the ERK signaling pathway to up-regulate pro-survival genes BCL2, tumor necrosis factor (TNF), nuclear factor κB (NF-κB), and inhibitor of NF-κB (I-κB) alpha to promote cell survival^[117]^. This pro-survival effect via ERK-BCL2 signaling on HCC cells is consistent with similar actions of CPE in neurons, mediated through binding to HTR1E receptor^[124]^, also present in HCC cells^[120]^. However, exogenously applied recombinant CPE had no effect on HCC cell proliferation and no change in the expression of cell cycle regulatory genes^[115]^. In contrast, when isolated sEVs secreted from high metastatic HCC cells which contain CPE protein were co-incubated with low metastatic HCC cells, they promoted proliferation and invasion of the cells^[112]^. sEVs isolated from high metastatic HCC cells treated with shCPE to suppress expression had no effect on promoting proliferation or invasion in low metastatic HCC cells, indicating this effect is dependent on sEV-CPE. The differential effects of soluble free CPE versus sEV-CPE on proliferation also support the presence of CPE in the sEVs, rather than being a contaminant in the EV preparation. Interestingly, when HEK293 cell sEVs loaded with CPE-shRNA were incubated with high metastatic HCC cells, these recipient cells exhibited downregulation in expression of CPE and cyclin D1, a cell cycle regulatory gene, and a decrease in proliferation. In summary [Figure 8], these HCC cell studies showed that CPE is trafficked to sEVs. Moreover, soluble CPE secreted from DCVs promoted stress-induced cell survival but not proliferation, while CPE in sEVs increased proliferation and invasion in recipient cells. These observations suggest that CPE released by the two different mechanisms may have distinct functions as mediators of intercellular communication.

### BDNF

In neurons, pro-BDNF and mature BDNF are packaged into classical secretory vesicles in the RSP and released upon stimulation into the synaptic cleft^[32]^. BDNF binds to tropomyosin receptor kinase B (TrKB) receptors in the post-synaptic neurons to activate various signaling pathways that mediate functions such as neurodevelopment, neuroprotection, and synaptic plasticity^[125-127]^. In addition, BDNF is also expressed in the gut and other tissues and plays a role in energy homeostasis including regulating glucose metabolism to prevent β-cell exhaustion^[128]^. Recently, BDNF has been found in sEVs isolated from the umbilical cord blood of neonates. The level of sEV BDNF was inversely correlated with cord ferritin levels and maternal iron deficiency^[129]^. In contrast, plasma BDNF was reported to be lowered in the umbilical cord blood of neonates from mothers with iron deficiency^[130]^. This difference indicates that BDNF associated with sEVs is present in the organelles and not due to plasma contamination. Iron deficiency in neonates is associated with neurodevelopmental impairment. Umbilical cord sEV BDNF can serve as a potential marker for iron deficiency and neonatal brain status, and may have a role in neuroprotection.

In another study, the level of mature BDNF in serum-derived sEV was found to be decreased and pro-BDNF increased in patients with Major Depression (MD) compared to controls^[111]^. This is of significance since the pro- and mature forms have opposite biological effects. Mature BDNF promotes neuronal survival and synaptic plasticity, while pro-BDNF binds to the p75 receptor and causes apoptosis^[131]^. Interestingly, the BDNF/pro-BDNF ratio was lower in sEV than in the serum of MD patients, suggesting different amounts of BDNF and pro-BDNF in these two compartments.

In a recent study^[116]^ using a newly developed NeuroDex ExoSort kit that employs immunoaffinity capture on magnetic beads with antibodies against growth-associated protein 43 (GP43) and Neuroligin (NLGN3), highly enriched neuron-derived EVs (NDEVs) were isolated from the plasma of control and Alzheimer Disease (AD) patients. Pro-BDNF was found to be much higher in the NDEVs than in the plasma of both control and AD patients, supporting the enrichment of this cargo in NDEVs. Interestingly, NDEV-associated proBDNF was reduced in AD versus control patients.

These studies provide examples of the RSP protein, BDNF, being trafficked and secreted from both classical secretory vesicles and sEV. The difference in the relationship of sEV BDNF versus plasma levels with iron deficiency, and changes in the forms (pro- or mature form) in the sEVs with psychiatric conditions, support pro-BDNF/BDNF being cargo in sEV. Furthermore, these studies suggest that circulating sEV pro-BDNF/BDNF can potentially serve as biomarkers for disease pathology, and may act as mediators of intercellular communication, delivering pro-BDNF/BDNF to other tissues. Pro-BDNF/BDNF as cargo in sEVs may serve to transport these molecules to distant targets in the periphery since they will be better protected from degradation in circulation.

### PTHrP

PTHrP is a 36 amino acid neuroendocrine peptide and survival factor and is synthesized by proteolytic cleavage from a prohormone, packaged into classical secretory vesicles (DCVs) and secreted via the regulated secretory pathway [Figure 1]. PTHrP is secreted from parathyroid cells, adrenal medulla, pituitary, pancreatic islets and the central nervous system, and in cancer cells. It is also secreted from osteoblastic cells and keratinocytes via the constitutive pathway [Figure 1]. PTHrP acts as a hormone by binding to a receptor, and some of its functions include stimulating adipocyte lipolysis^[132]^, regulating calcium homeostasis, and recruiting osteoblasts for bone formation^[132]^. PTHrP is another example of a RSP protein found in sEVs. sEVs derived from Lewis Lung carcinoma (LLC) containing PTHrP have been shown to induce lipolysis in murine 3T3-L1 adipocytes^[110]^. Condition medium from LLC cells exposed to Rab27A shRNA to inhibit EV release had a lower effect on lipolysis than that from control cells, confirming that LLC-EVs contribute to lipolysis. These LLC EVs fused directly with 3T3-L1 cells and were internalized in a caveolin /raft-mediated manner, transferring PTHrP to the recipient cells to induce lipolysis. This lipolytic activity was blocked by the presence of PTHrP neutralizing antibody. After centrifugation, the supernatant fraction of medium containing PTHrP secreted from constitutive secretory vesicles from LLC cells or recombinant PTHrP stimulated lipolysis when incubated with 3T3-L1 cells, indicating a dual mechanism of PTHrP release from the LLC cells. Knocking down PTHrP receptor expression in 3T3-L1 cells eliminated the lipolytic activity induced by LLC-derived sEVs, suggesting that the effect is dependent on extracellular PTHrP receptor. Since this study has shown that the LLC-derived EVs containing PTHrP are fused with the 3T3-L1 cells and internalized, the mechanism by which the sEV cargo, PTHrP, is able to get recycled and secreted to get exposure to the extracellular receptor to mediate its activity is unclear. Nevertheless, the data presented do support the release of PTHrP by a dual mechanism via secretory vesicles and in sEVs. In this case, PTHrP from the two secretory mechanisms performed a similar function. One can suggest that the delivery of PTHrP in the circulation to target cells at a distance would be better protected from degradation inside EVs.

### Other RSP proteins trafficked to sEVs

With new proteomic data emerging for sEV, more RSP cargo proteins have been found to be associated with these organelles. Among these is APP, which is synthesized in neurons and astrocytes. In neuron-like chromaffin cells, APP is processed to β-amyloid peptides (Aβ1-40 and Aβ1-42), which are co-secreted with peptide neurotransmitters (Galanin) and catecholamines from DCVs upon stimulation. APP, along with secretases that convert APP into Aβ peptides, were present in purified DCVs. β-Amyloid peptides have also been reported to be released from sEV^[109,116]^. Aβ1-42 levels in immunoaffinity-purified NDEVs from plasma have been shown to be higher in AD patients compared to controls^[116]^.

Another example is the granins which are trafficked into DCVs and released in a regulated manner^[15,16]^. Some of these granins are processed into bioactive peptides. Proteomic analysis revealed CgA in the sEV of human-induced pluripotent stem cell neurons expressing mutant presenilin 1. However, CgA was not in the sEV of control cells^[113]^. This suggests that CgA may be trafficked to sEV under certain pathological conditions.

## CONCLUDING REMARKS AND FUTURE DIRECTIONS

Here, we have summarized advancements in the molecular mechanisms for prohormone/neurotrophic factor sorting, packaging, and transport in secretory vesicles for stimulated release from endocrine cells and neurons. Recent studies have revealed that RSP proteins (examples above) are also trafficked to extracellular vesicles/sEVs in neuroendocrine and cancer cells. Moreover, in one example, CPE/NF-α1, the physiological functions may be different depending upon whether it is released from classical secretory vesicles (DCVs) or in sEVs. It will not be surprising if RSP proteins within sEVs have different intracellular roles after they are released from internalized sEVs into the cytoplasm/nucleus of recipient cells. More studies in the future will illuminate this possibility for other RSP proteins. Another advantage of transporting these RSP protein/peptide molecules in sEVs to distant targets for intercellular communication may be to increase their half-life in circulation since sEVs are stable and the cargo can be protected from degradation. This may possibly expand the functions of such hormones or neurotrophic factors as they reach more distant targets. It is likely, as more studies are done, that many other peptide hormones, neurotrophic factors, and regulated secretory proteins could be found in EVs. This new concept is in its infancy and many questions remain unanswered.

Most critical is how the hormones and neurotrophic factors, including their precursors, get packaged into EVs/sEVs. Based on the discovery of Nobel Laurette Gunter Blobel^[133]^, these hormones and neurotrophic factors have an N-terminal signal peptide that directs them into the RER cisternae and into the secretory vesicle pathway. One possibility is that RSP proteins may go into vesicles formed by budding from the TGN, which then fuse with endosomes. Transmembrane cargo, such as the transmembrane form of CPE/NF-α1, would then be retained on the endosome until it matured into a MVB, which would allow invagination and ILV formation. Since transmembrane CPE/NF-α1 is a sorting receptor to which prohormones and proBDNF bind tightly at an acidic pH, they may stay associated and ride with CPE/NF-α1 into ILVs. Another possibility is that RSP proteins, after secretion, could be endocytosed and end up in endosomes and then in ILVs. Alternatively, ILVs could be formed through the endoplasmic reticulum (ER)/Golgi secretory pathway, packaging RSP proteins in them during the process. The ILVs are then taken up by MVBs and released as sEV or extracellular vesicles [Figure 1]. A recent report studying the prostate-like Secondary Cell (SC) of Drosophila male accessory gland demonstrated the presence of Rab6 positive-DCVs and -ILVs within the same compartments derived from the TGN. Then Rab 6 in the membrane in these organelles is transitioned to Rab11 for both Rab11 -exosome and DCV biogenesis, indicating that these two processes may be interdependent^[134]^. Indeed, Rab GTPases are known to control many aspects of vesicle trafficking by acting as regulatable switches^[135]^. While the SCs are a specialized secretory system, these observations indicate the sharing of communication between the RSP and the endosomal systems for DCV and EV biogenesis and hence the possible packaging of RSP cargo into sEVs. More studies on mammalian secretory cells will determine if this communication between the RSP and endosomal system also occurs.

Another important question is, since these hormones and neurotrophic factors are packaged with other specific components such as RNA and microRNA in EVs, how is that accomplished? Are they randomly or selectively co-packaged? Finally, although it is clear that the hormones or neurotrophic factors in the sEVs are taken up by recipient cells and exert specific physiological effects as reflected by changes in the cell’s mRNA and microRNA profiles, the details of the mechanism of action remain elusive. Many more cell biological studies in the future are necessary to shed light on these questions. It is hoped that our inclusion of a review of the molecular players involved in the intracellular trafficking of hormones to the RSP will inspire more cell biologists to study the molecular mechanisms in detail for the packaging of RSP proteins into EVs/sEVs. Furthermore, the secretion, uptake, and internalization of these EVs and the physiological function of the internalized hormones and neurotrophic factors in the recipient cells warrant exploration.
